# Exploring Personal Exposure to Airborne Microplastics across Various Work Environments in Pathum Thani Province, Thailand

**DOI:** 10.3390/ijerph20247162

**Published:** 2023-12-11

**Authors:** Kanokwan Limsiriwong, Ekbordin Winijkul

**Affiliations:** Environmental Engineering and Management, Asian Institute of Technology, Pathum Thani 12120, Thailand; kanokwan@ait.asia

**Keywords:** personal exposure, airborne microplastics, personal air sampler

## Abstract

This study used personal air samplers to assess the concentration of airborne microplastics exposed by different occupational groups during their working hours. The personal air sampler was placed in the “breathing zone” of the worker during working hours to collect microplastic exposure data. Occupations examined included housekeepers, laundromat staff, office workers, van drivers, street vendors, maintenance technicians in wastewater treatment plants, and waste segregation officers in the university and market. The level of airborne microplastic exposure was found to be influenced by the daily activities and environmental conditions of the workplace. Waste segregation officers in the university and market exhibited the highest levels of exposure to airborne microplastics, at 3964 ± 2575 microplastics per cubic meter (n/m^3^) and 3474 ± 678 n/m^3^, respectively. Further analysis focused on airborne microplastics less than 10 µm in size which can be taken in through inhalation. During the 8 h working period, the waste segregation officer in the university recorded the highest 10 µm airborne microplastic intake, at 5460 pieces, followed by the waste segregation officer in the market at 3301 pieces, housekeepers at 899 pieces, van drivers at 721 pieces, maintenance technicians in WWTPs at 668 pieces, laundromat staff at 454 pieces, street vendors at 249 pieces, and office workers at 131 pieces.

## 1. Introduction

Microplastics, ranging in size from 1 µm to 5 mm, present a significant challenge due to their persistence in the environment and potential adverse impacts on both human health and the well-being of our ecosystems [1]. There are two main types of microplastics. The first type is primary microplastics, which are manufactured and discharged directly into the environment. The second type is secondary microplastics, which are generated when larger materials, such as textiles, tires, personal care products, and mismanaged urban plastic waste, break down or degrade. 

Microplastics are present in various environmental pathways, including marine ecosystems, freshwater, and terrestrial systems [2,3]. The atmosphere is one of the pathways in which microplastic pollutants exist as it is evident that microplastic fibers have been found in outdoor and indoor air [4,5]. Thus, humans are at risk of exposure to airborne microplastics through breathing the air. Depending on their size, airborne fibrous microplastics have a chance of entering our respiratory system. Some microplastics can evade the mucociliary clearance mechanisms of the lung, and they can be deposited in the respiratory system, particularly in people with impaired clearance mechanisms [4]. Additionally, there is supporting evidence that all polymeric particles are smaller than 5.5 µm in size, and fibers with lengths ranging from 8.12 to 16.8 µm have been observed in human lung tissue [6].

Moreover, airborne microplastics, particularly in urban areas where metals and polycyclic aromatic hydrocarbons (PAHs) are generated from various emission sources, can absorb toxic pollutants on their hydrophobic surfaces [7]. These substances have the potential to be harmful to human health, causing mutations, carcinogenic effects, and reproductive toxicity [4]. Additionally, airborne microplastics themselves possess physical mechanisms that can lead to toxicity in humans [8]. Studies on the effects of microplastics on the human body are limited, but exposure to high concentrations of plastic particles has been linked to occupational diseases in industrial workers. Prata (2018) investigated diseases found in workers from three types of industries—synthetic textile, flocking, and polyvinyl chloride industries—and reported symptoms among workers such as throat irritation, shortness of breath, cough, and chest pain. These symptoms, along with signs of chronic inflammation and irritation, may lead to lung fibrosis or even cancer [9,10].

In recent years, the number of publications on atmospheric microplastic monitoring has increased, and diseases among workers near sources of microplastics have been reported. Environmental exposure to airborne microplastics has not been studied extensively, and there is limited research on the quantity of inhaled microplastics in the human body. Thus, this study used a personal air sampler to collect airborne microplastic samples and assessed personal exposure to airborne microplastics at different workplaces in Pathum Thani, Thailand.

## 2. Materials and Methods

### 2.1. Sample Groups

**Figure 1:**
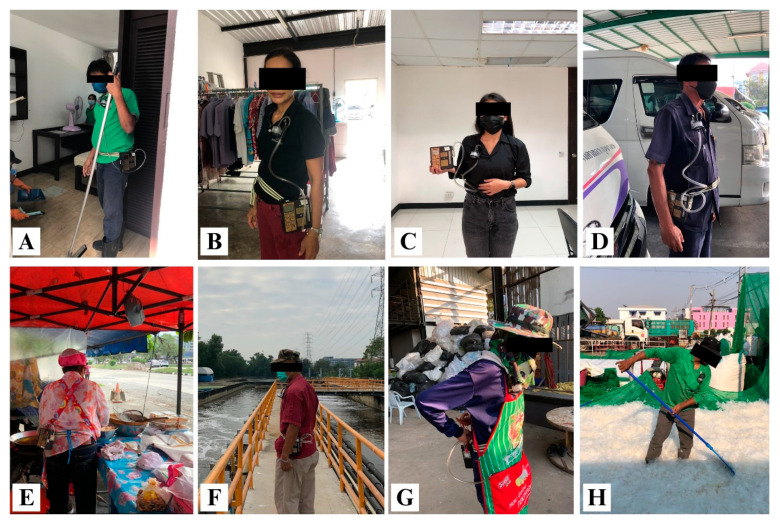
Sample groups in this study: (**A**) housekeeper; (**B**) laundromat staff; (**C**) office worker; (**D**) van driver; (**E**) street vendor; (**F**) maintenance technician in a WWTP; (**G**) waste segregation officer at the university; (**H**) waste segregation officer in the market.

This study evaluated the levels of airborne microplastics to which workers were exposed in various workplace settings. These workplaces were separated into indoor and outdoor environments. In total, eight workplaces were selected, representing common daily working locations, i.e., roadside, waste separation plants (WSPs), buildings, dormitories, and wastewater treatment plants (WWTPs). The sample groups in indoor environments comprised housekeepers, laundromat staff, office workers, and van drivers. On the other hand, the sample groups in outdoor environments included street vendors, maintenance technicians in WWTPs, waste segregation officers at the waste separation plant in the university, and waste segregation officers at the waste separation plant in the market. In this context, three samples were collected for each sample group. Figure 1 shows photographs of each sample group in this study, and the number of samples for each group is provided in Table 1. 

### 2.2. Sampling Method

**Figure 2:**
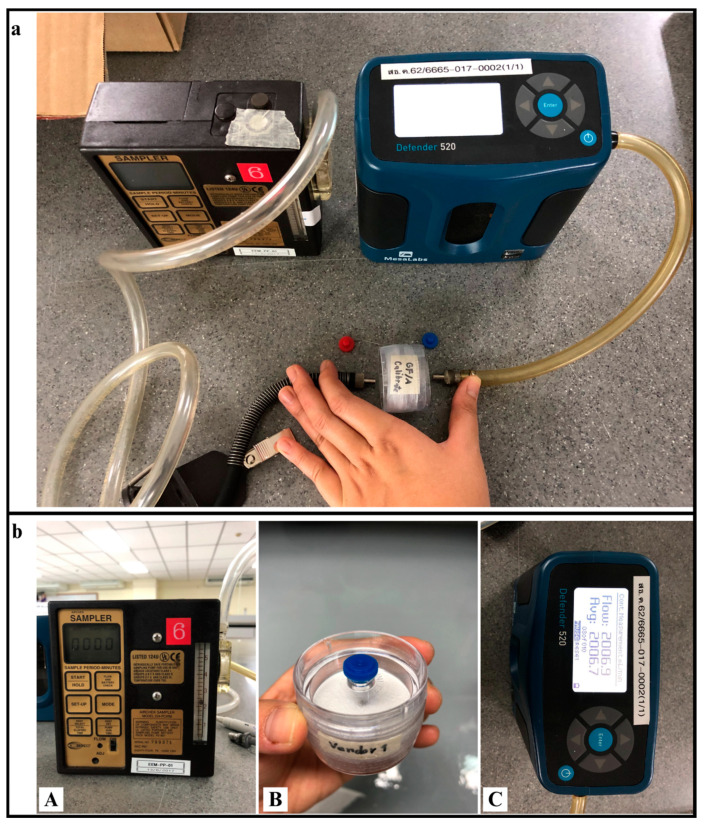
(**a**) The personal air sampler; (**b**) component of the personal air sampler: (**A**) personal air sampling pump; (**B**) GF/A pad placed in cassette filter; (**C**) pump calibration equipment.

Personal air samplers were used for the total suspended particle (TSP) exposure study, following the sampling method proposed by NIOSH0500. The airflow rate of the device was set at 2 L/min. All microplastic samples for different occupations were collected for 8 h (including lunchtime), following the NIOSH recommended time average to collect a sample [11]. For the reliability of the data collection among different sample groups, this study collected samples three times from each occupational group (on different days of the week in the same week). Additionally, all samples in this study were collected during the same period (dry season), during December 2022 and February 2023 (detailed information on the sample groups is available in the Supplementary Materials, Tables S1–S8). The sampling device and GF/A with micropore 1.6 µm (Figure 2) was set in the “breathing zone” of the worker. The weight of the filters was recorded before and after sampling. The concentration of TSP was calculated based on the weight of total mass per volume of air (Equation (1)). After collection, the samples were placed in a desiccator at room temperature for preservation before proceeding with staining using the Nile Red method.

(1) $$C=\frac{W\times 1000}{V}$$

where *C* is the concentration of TSP in mg/m^3^, *W* is the weight of total particles on the filter in mg, and *V* is the volume of sampling air in m^3^.

### 2.3. Microplastic Identification Method

#### 2.3.1. Nile Red Staining

The Nile Red staining technique (NR-S), involving the use of Nile Red (NR) dye to stain polymer particles, is a widely employed method for analyzing airborne microplastics (AMPs) in environmental samples [12,13,14]. It proves helpful in identifying concealed microplastics and serves as a valuable preliminary step before further analysis using a fluorescence microscope. To prepare the Nile Red, the initial step involves mixing 1 mg of Nile Red with 1 mL of Chloroform [12]. The solution is then filtered through a syringe filter (0.22 µm) into a clean glass screw-top vial (20 mL) [15,16]. To preserve Nile Red stock solution, aluminum foil is used to cover the sample, and it is kept in the freezer at −4 °C. For the second step, 0.1 mL of Nile Red stock solution is diluted with 20 mL distilled water (resulting in a Nile Red concentration of 5 µm/mL for microplastic staining) [15], and the solution should not be kept overnight [16]. The amount of Nile Red liquid to be added on the sample (0.7–2.5 mL) depends on the organic material content of the sample [13]. The sample is then dried in glass Petri dishes at 60 °C in the oven before being placed in a desiccator [17], covered with aluminum foil, and stored in a dark room.

#### 2.3.2. Microplastic Detection by Fluorescence Microscopy and ATR-FTIR Spectroscopy

Fluorescence microscopy is a visual identification method; it was used to identify the shape and size of microplastics after staining with Nile Red. In this study, a fluorescence microscope (Olympus SZX16, Tokyo, Japan) with a 3.2× objective lens was used to inspect the microplastic in four size ranges (<10 µm, 10–100 µm, 100–1000 µm, and >1000 µm). The images of the microplastics on the sample were captured using a camera (Olympus DP73, Tokyo, Japan), and Olympus CellScens Standard software was used to measure the length of the microplastics. Subsequently, the number concentration of airborne microplastics was calculated using Equation (2).

(2) $$\mathrm{Number}\;\mathrm{concentration}=\frac{N}{V\;}$$

where Number concentration is the number of microplastics/m^3^, *N* is the number of microplastics on the filter (number), and *V* is the volume of the sampling air (m^3^).

ATR-FTIR spectroscopy was used to identify the types of microplastics on the filters. The polymer type was determined by identifying the polymer with the strongest match in the FTIR database. For each sample group, the filter sample with the higher concentration of microplastics was selected for FTIR analysis, and five points in each sample were chosen for the analysis. A Fourier Transform Infrared Microscope (Nicolet iN10, Thermo Scientific, Waltham, MA, USA), equipped with Cooled (MCT-A Detector) and Thermo Scientific OMNIC™ Picta™ software, was used to inspect and match types of microplastics in this study.

### 2.4. Results and Evaluation

Statistical analysis was used to evaluate and compare the correlation between the amount of airborne microplastic and the concentration of TSP in all sample groups, including the correlation between the amount of airborne microplastic and metrological parameters in all sample groups. In this study, Pearson correlation was performed in SPSS to analyze the data.

## 3. Results

### 3.1. Concentration of AMPs in Different Occupational (Sample) Groups

**Figure 3:**
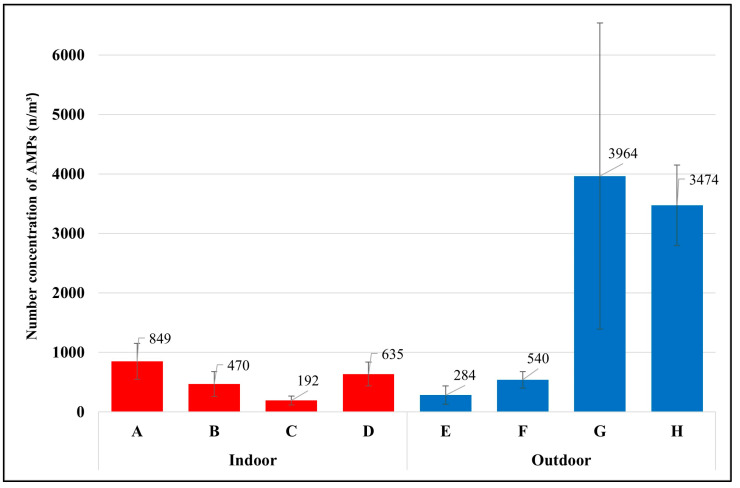
Number concentration of AMPs in each occupational group (mean ± S.D.); (A) housekeeper, (B) laundromat staff, (C) Office worker, (D) van driver, (E) street vendor, (F) maintenance technician in the WWTP, (G) waste segregation officer in the university, (H) waste segregation officer in the market.

This study found that each occupation had different exposures to airborne microplastics depending on daily activities and environmental conditions of the workplace. The average temperature of all sample groups was 25 ± 3.7 °C, with a maximum of 31 °C and a minimum of 18 °C (detailed information on the sample groups, daily activities, and environmental conditions of the workplace are available in the Supplementary Materials, Tables S1–S8). The highest level of exposure to airborne microplastics was found with the waste segregation officer in the university, with 3964 ± 2575 microplastics per cubic meter (n/m^3^), and the waste segregation officer in the market (3474 ± 678 n/m^3^), followed by the housekeeper (849 ± 303 n/m^3^), van driver (635 ± 199 n/m^3^), maintenance technician in WWTPs (540 ± 136 n/m^3^), laundromat staff (470 ± 206 n/m^3^), street vendor (284 ± 155 n/m^3^), and office worker (192 ± 73 n/m^3^), as presented in Figure 3.

The two groups of waste segregation officers had the highest exposure to airborne microplastics because of their daily activities of working to process plastic waste. These workers were working very close to the plastic blender (airborne microplastic source). For example, the waste segregation officer at the market is responsible for segregating the types and colors of plastic trays before crushing them into small sizes with a blender. The plastics are then laid on the ground to dry. The officer turns the plastic bags from the bottom to the top to ensure they are dry before sending them for the recycling process. Generally, plastic polymers in the environment take a very long time to degrade via natural accelerants [18]. Considering that waste segregation officers were directly exposed to the source of microplastics and should not be combined with other occupations in the outdoor environment, the average exposure of the outdoor occupations without waste segregation officers became 412 n/m^3^, which was lower than the average exposure of the indoor occupations (536 n/m^3^).

Beyond the daily activities and environmental conditions that influence the concentration of AMPs, this study also documented ambient air conditions, including temperature, humidity, and pressure (detailed information on the environmental condition of the workplace is available in Supplementary Materials, Tables S1–S8). Pearson correlation (r) was employed to assess the relationship between AMPs and environmental conditions. A weak positive correlation (r = 0.33) was observed between the number concentration of airborne microplastics and temperature in all samples, while a weak negative correlation (r = −0.12) was found between the number concentration of airborne microplastics and humidity in all samples. Additionally, there was a notably weaker negative correlation or no discernible relationship between pressure and AMPs in all samples (r = −0.02). However, it is important to note that this study recorded environmental conditions only at the start of each sample collection. Therefore, more samples and environmental conditions are needed to further investigate the effects of environmental conditions on the number concentration of AMPs.

### 3.2. Shapes of AMPs at Difference Workplaces

**Figure 4:**
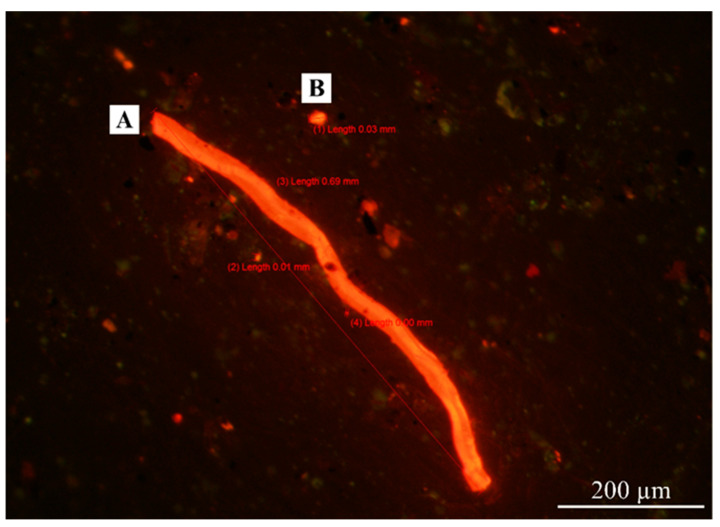
Shapes of Nile-Red-stained AMPs inspected by fluorescence microscopy with a 11.5× objective lens. (A) Fiber; (B) fragment.

**Figure 5:**
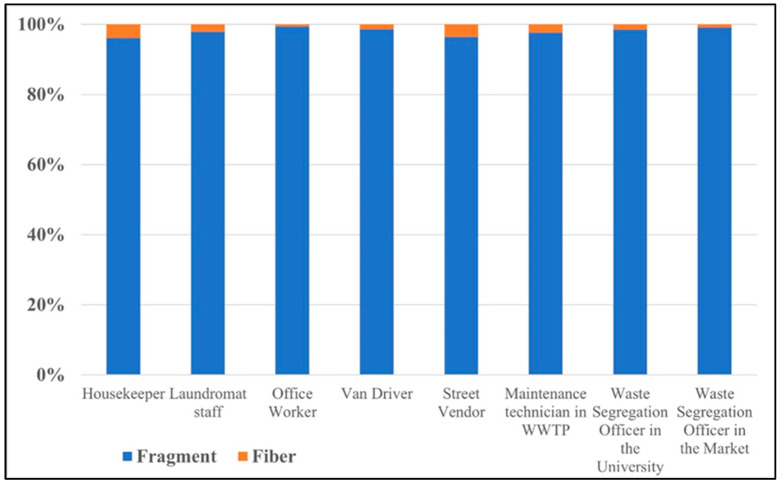
Percentage of different AMP shapes in each occupational group.

The World Health Organization (WHO) defines a fiber as any particle that has a length > 5 µm with a diameter < 3 µm and an aspect (length-to-diameter) ratio > 3:1 [19]. The fiber shape definition from WHO was employed to identify fiber shapes under the fluorescence microscope, making it possible to compare the results of this study with other studies. However, the light scattering of microplastic particles under the microscope posed a challenge in classifying plastic shapes other than fibers. As a result, other plastic shapes were categorized as fragments, as shown in Figure 4. Figure 5 shows the shapes of AMPs in different occupational groups in this study.

From Figure 5, all occupation groups have the potential to inhale microplastics in a fragment shape, ranging from 96.2% to 99.5% of the total number of airborne microplastics. These fragment microplastics are expected to be secondary microplastics resulting from plastic material degradation or microplastic fragmentation [20,21]. The fiber-shape microplastics accounted for 0.5% to 3.8% of the total airborne microplastics, which may have originated from synthetic textiles or fabric tearing [4,22].

### 3.3. Size of AMPs in Difference Workplaces

**Figure 6:**
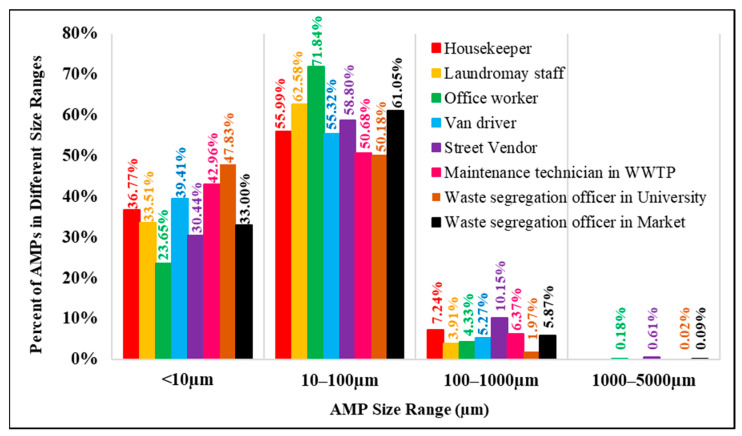
Percent of AMPs in different size ranges in each occupational group.

Figure 6 shows that airborne microplastics with a size of 10 µm–100 µm dominated the total microplastics in our study; all workers were exposed to microplastics in this size range, accounting for 50.2% to 71.8% of all AMP exposure. The second rank is airborne microplastics with a size of less than 10 µm, to which different groups are exposed from 23.6% to 47.8% of total AMP exposure. Microplastics with a size < 10 µm should be a concern for the impacts on human health because they can escape the mucociliary clearance mechanisms of the lung and can be deposited in the respiratory system. Amato-Lourenço et al. (2021) found that microplastics < 5.5 µm and fibers of sizes of 8.12–16.8 µm have been observed in human lung tissue [6]. 

Microplastics larger than 100 µm accounted for less than 10% of all AMP exposure during working hours, and should involve less health concern since they can be removed by self-cleaning mechanisms of the human body and deposition by Earth’s gravity. However, caution should be exercised when workers are exposed to sizable microplastics in close proximity to the sources of airborne microplastics.

### 3.4. Type of AMPs at Difference Workplaces

In this study, ATR-FTIR spectroscopy was used to identify microplastic types in the samples from each occupational group. The limitation of ATR-FTIR spectroscopy is that the minimum size of plastic that can be detected is approximately 10 µm, and the accuracy is significantly reduced for samples sized < 50 µm [23,24,25].

For an indoor environment, microplastics were identified as polyethylene terephthalate (PET), polyester (PES), styrene acrylonitrile copolymer (SAN), and cellophane. The sample collected from the housekeeper was identified as PET and PES. The sample collected from Laundromat staff was identified as SAN. The sample collected from the office worker was cellophane. The plastic types of SAN, PES, and cellophane were detected in the van driver sample. 

More types of airborne microplastics were detected in the outdoor samples than in the indoor samples. Seven types of polymers were found in the outdoor areas as polyethylene (PE), polypropylene (PP), polystyrene (PS), ethylene-vinyl acetate (EVA), PET, PES, and SAN. PET, PES, and SAN are general polymers that were detected in both environments. Microplastic types in the sample collected from the street vendor were PES and PE. Airborne microplastics collected from a maintenance technician in WWTP were identified as PET. PP was a polymer type found in the sample of the waste segregation officer at the university. Various microplastic types (PS, EVA, SAN, and PET) were found in the sample collected from the waste segregation officer in the market.

### 3.5. Concentration of TSP in Each Workplace

**Figure 7:**
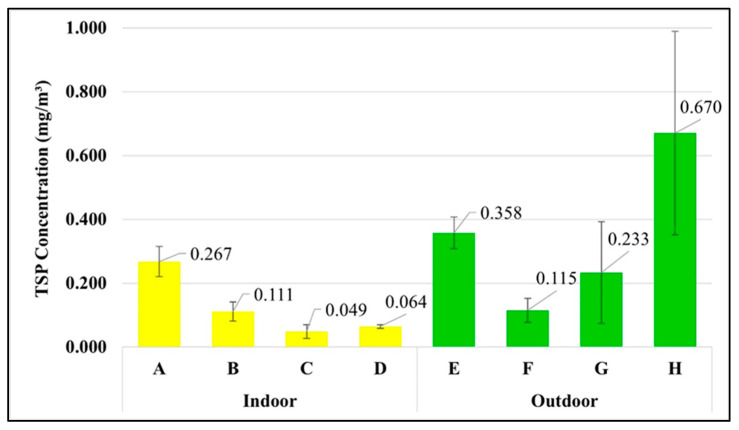
Concentration of TSP in each occupational group (mean ± S.D.): (A) housekeeper, (B) laundromat staff, (C) office worker, (D) van driver, (E) street vendor, (F) maintenance technician in the WWTP, (G) waste segregation officer in the university, and (H) waste segregation officer in the market.

Microplastics are atmospheric particles. Unlike TSP, there is no standard for airborne microplastics in the atmosphere and the workplace. Figure 7 shows that the waste segregation officer in the market has the highest exposure to TSP concentration at 0.670 ± 0.319 mg/m^3^, followed by the street vendor (0.358 ± 0.049 mg/m^3^) > housekeeper (0.267 ± 0.047 mg/m^3^) > waste segregation officer in the university (0.233 ± 0.159 mg/m^3^) > maintenance technician in the WWTP (0.115 ± 0.038 mg/m^3^) > laundromat staff (0.111 ± 0.030 mg/m^3^) > van driver (0.064 ± 0.006 mg/m^3^) > office worker (0.049 ± 0.022 mg/m^3^). The Occupational Safety and Health Administration (OSHA) proposed the restrictions for air contaminants [26] in the form of permissible exposure limits (PELs) during the time weight average of 8 h for the TSP as 15 mg/m^3^. Therefore, the TSP exposures in different occupational groups in this study complied with the OSHA standards.

### 3.6. Comparison of AMP and TSP Concentrations at Difference Workplaces

**Figure 8:**
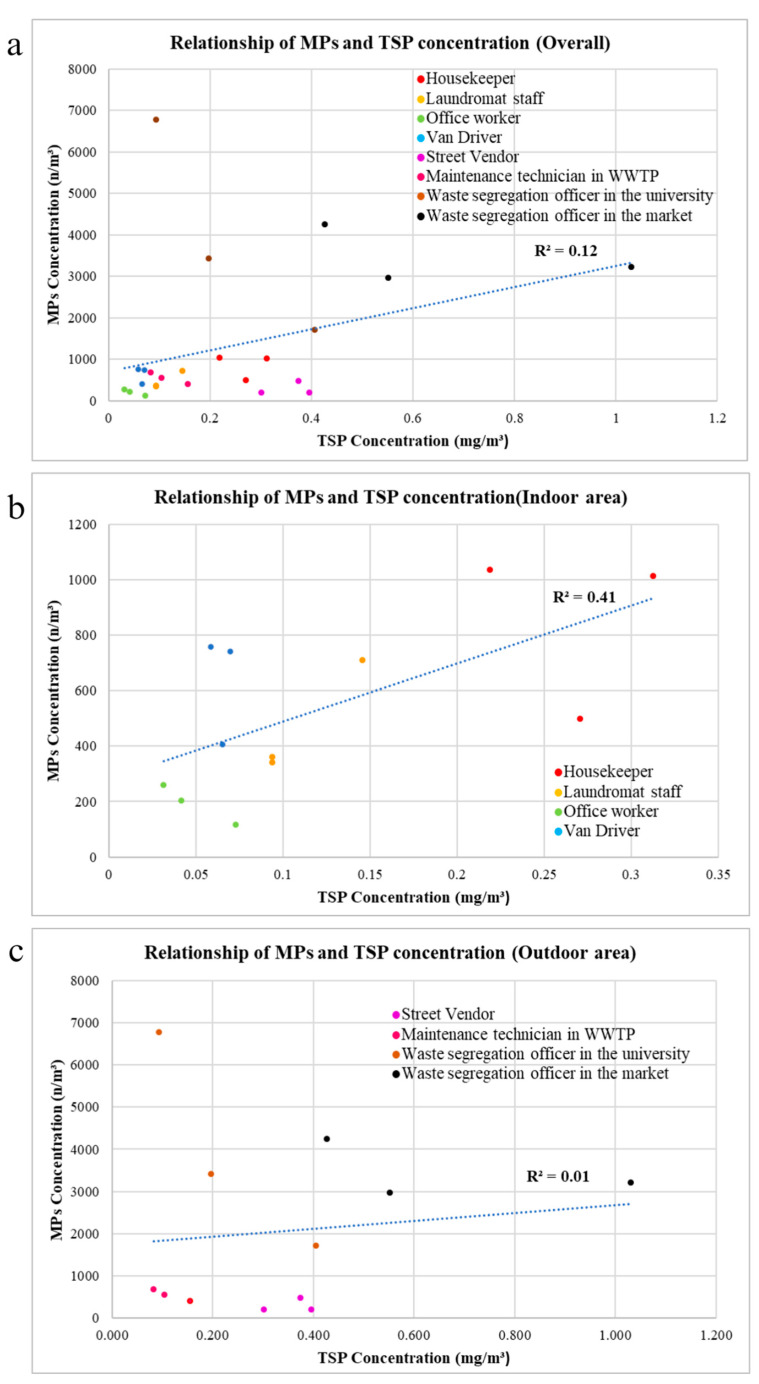
Scatter plot of TSP concentration and AMP number concentration: (**a**) all samples; (**b**) indoor conditions; (**c**) outdoor conditions. Different color dots refer to different sample groups.

The relationship between TSP and number of microplastics from all 24 samples, collected from 8 occupational groups, is shown in Figure 8a. There is a weak positive correlation (R^2^ = 0.12) between the TSP concentration and the number concentration of microplastics, meaning that an increase in TSP concentration may lead to a higher chance of detecting more microplastics. In addition, Pearson correlation showed that TSP concentration and airborne microplastic number concentration have a statistically significant correlation at 0.347 (n = 24) and Sig. (2 tailed) = 0.097.

In the indoor area, the relationship between TSP and the number concentration of microplastics in all 12 samples collected from four occupational groups is depicted in Figure 8b. A positive correlation (R^2^ = 0.41) exists between the TSP concentration and the number concentration of AMPs. Furthermore, the relationship between TSP and the number of AMPs in the outdoor area, based on all 12 samples collected from four occupational groups, is illustrated in Figure 8c. This is also a positive correlation (R^2^ = 0.01), but it is notably weaker compared with the indoor area. 

In outdoor environments, there are various sources of TSP, such as road dust, industrial activities, power plants, and domestic burning, but the primary contributors to the release of AMPs are plastic particles resulting from human activities, undergoing physical and chemical degradation before becoming airborne [14,26]. Additionally, the fate of microplastics in outdoor atmospheric environments is influenced by various factors, such as wind speed, wind direction, precipitation, and temperature [27]. Thus, this may be the reason for the lower correlation between TSP and microplastics in the outdoor environment (where more TSP sources are available) than the correlation in the indoor environment.

## 4. Discussion

The results of the number of airborne microplastic exposures in each occupational group in this study cannot be compared with other studies due to the absence of prior research on airborne microplastic exposure. However, four previous studies that sampled microplastics in the air were selected for magnitude comparison. In addition, another study using a breathing thermal manikin to measure microplastic exposure during males’ light activity was also selected (Table 2).

Regarding the characteristics of airborne microplastics (AMPs), previous studies have consistently shown that the majority of microplastics in the atmosphere are smaller than 100 µm. More than 90% of AMPs in the outdoor and indoor environment, with sizes less than 30 µm and 100 µm, were detected in China [28,29] respectively. In Thailand, airborne microplastics with sizes between 10 and 100 µm and less than 10 µm were found at rates of 51.9%, and 46.9%, respectively [5]. In this study, similar findings were observed, with at least 90% of the exposures involving AMPs smaller than 100 µm. 

Comparing with previous research, the airborne microplastic concentration in the outdoor environment in five megacities (China) was 282 ± 127 n/m^3^ [28]. AMP concentrations in the indoor and outdoor areas in Wenzhou, China, were 1583 ± 1180 n/m^3^ and 189 ± 85 n/m^3^, respectively [29]. Research in Thailand provided the concentration of microplastics in the outdoor areas at five locations, ranging from 201.72 ± 15.58 n/m^3^ to 581.90 ± 28.39 n/m^3^ [5]. For the experiment with a breathing thermal manikin, the airborne microplastic concentration that a male person exerting light activity can be exposed to through inhalation was 9.3 ± 5.8 n/m^3^, or around 272 n/day [9]. 

In this study, the lowest airborne microplastic exposure was found in the office worker group, at 192 ± 73 n/m^3^, while the highest exposure was found in the waste segregation officer at the university, at 3964 ± 2575 n/m^3^. The range of microplastics found in this study was higher compared with those found in previous studies. Several factors may contribute to this difference:

- First, this study used an 8 h working time to collect sample. This may have led to the higher concentration in our study than the sampling performed over 24 h in other studies.
- Second, the working period may be the time when airborne microplastics are generated. The research in China presented the concentration of AMPs in five megacities; the concentration peaked at noon, followed by morning and night [28].
- Third, the samples collected from the personal air samples attached to the workers may collect at a closer distance to the sources that generate airborne microplastics, such as solid waste separation plants, than the ambient airborne microplastic concentrations in other studies.

**Figure 9:**
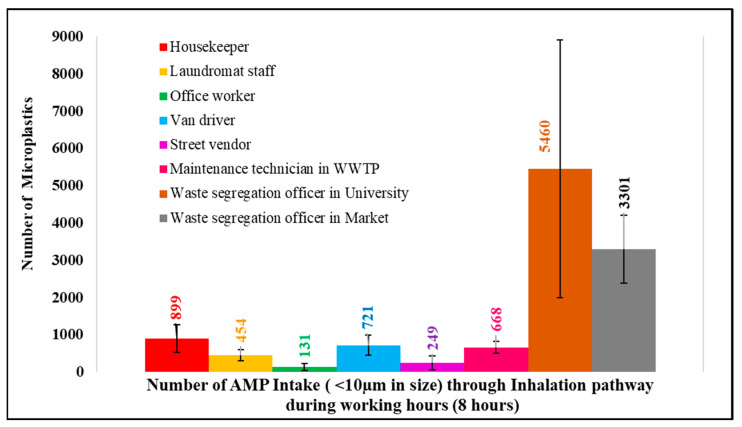
AMP intake (<10 µm) via the inhalation pathway during working hours.

To estimate microplastic intake through inhalation pathways during working hours, the human breathing rate of 6 L/min [10,30] was applied. In addition, only microplastics smaller than 10 µm were considered for this calculation, as these particles are breathable and can deposit in the deep respiratory system, potentially causing impacting on health [10,31,32]. Therefore, during working hours, each occupation has a potential airborne microplastic intake (in numbers), as shown in Figure 9.

The results show that the potential microplastic intake (size < 10 µm) during the 8 h working period ranged from 131 to 5460 microplastics through inhalation. However, the microplastic intake through inhalation remained minor compared with ingestion (Table 3).

From Table 3, microplastic intake through ingestion (80.19 × 10^6^ microplastics/day) [33] was much higher than microplastic intake through inhalation (131–5460 microplastics/8 h; or simply, multiply by three to make 24 h as 26.97 × 10^2^–16.38 × 10^3^ microplastics/day). 

However, it is evidenced that airborne microplastics align with the epidemiology of occupational disease [10]. Hence, this study recommends that workers should wear protective equipment to avoid breathing airborne microplastics during working hours, such as N95 masks [37].

## 5. Conclusions

This study used a personal air sampler to measure the concentrations of airborne microplastics to which different occupations are exposed during their working periods. The occupational groups included in this study were housekeeper, laundromat staff, office worker, van driver, street vendor, maintenance technician in a WWTP, waste segregation officer in a university, and waste segregation officer in a market. The level of exposure to airborne microplastic depends on the daily activities and environmental conditions of the workplace. The waste segregation officers in the university and the market had the highest level of exposure to airborne microplastics, at 3964 ± 2575 n/m^3^ and 3474 ± 678 n/m^3^, respectively. 

However, when focusing on airborne microplastics (AMPs) less than 10 µm in size and calculating microplastic intake through inhalation, the waste segregation officer in the university had a microplastic intake of 5460 pieces during working time, followed by the waste segregation officer in the market (3301 pieces) > the housekeeper (899 pieces) > the van driver (721 pieces) > the maintenance technician in WWTPs (668 pieces) > the laundromat staff (454 pieces) > the street vendor (249 pieces) > the office worker (131 pieces).

Moreover, this study found that workers have higher exposure to fragment-shaped microplastics than fiber-shaped microplastics, and microplastics in the size range of 10–100 µm were most commonly found in the samples of all workers. In addition, four types of polymers, i.e., PET, PES, SAN, and cellophane, were found in the indoor area, while seven types of polymers, i.e., PES, PE, PET, PP, PS, EVA, and SAN, were found in the outdoor area. 

The microplastic intake through inhalation during working hours in this study ranged from 131 to 5460 pieces; this intake could be mitigated by the wearing of N95 masks during working periods.

## Figures and Tables

**Table 1:** Sample groups.

Working Condition	Target Group	Samples (n)
Indoor	Housekeeper	3
	Laundromat staff	3
	Office worker	3
	Van driver	3
Outdoor	Street vendor	3
	Maintenance technician in WWTP	3
	Waste segregation officer in university	3
	Waste segregation officer in Market	3
Total sample		24

**Table 2:** Comparison of AMPs with previous studies.

Sample	Preparation Method	Filter Type	Location	Number of AMPs (n/m^3^)	Characteristic of AMPs			Reference
					Shape	Size (µm)	Polymer Type	
AMPs (outdoor air, 24 h)	NR staining	PTFE filter	Beijing, China	393 ± 112	Fragment 88.2% and fiber 11.8%	<30 µm: 61.6%, 30–100 µm: 33.1%, 100–300 µm: 0.5%, 300–1000 µm: 0.5%, >1000 µm: 0.03%	PE, PS, PVC, PA, PP, and PET	[28]
			Tianjin, China	324 ± 145				
			Shanghai, China	267 ± 117				
			Hangzhou, China	246 ± 78				
			Nanjing, China	177 ± 59				
Total suspended particulate sampler (24 h)	Wet peroxide oxidation (WPO), NR staining	GF/F filter 0.7 µm	Wenzhou, China (Indoor)	1583 ± 1180	Fragments 83.5–94.2%, Fiber	5–30 µm; 54.1–65.2%, 30–100 µm; 25.3–32.8%, >100 µm; 11.0%	PES, PA, PE, PP, PS, PVC, and other	[29]
			Wenzhou, China (Outdoor)	189 ± 85	Fragments 89.7–96.3%, Fiber	5–30 µm; 58.9–72.3%, 30–100 µm; 24.6–33.7%, >100 µm; 5.5%		
The high-volume air sampler (outdoor air, 24 h)	NR staining	Quartz filter 2.2 µm	University, Pathum Thani	201.72 ± 15.58	Fragments: 97.22%, Fibers: 2.78%	2.2–2.5 µm: 19.89%, 2.5–10 µm: 27.09%, 10–100 µm: 51.94%, 100–300 µm: 0.65%, 300–1000 µm: 0.34%, 1000–5000 µm: 0.10%	PE, PP, and cellophane	[5]
			Roadside, Bangkok	349.53 ± 18.53			PE, PU, and cellophane	
			Urban Park, Bangkok	312.45 ± 50.43			PE, PU, and cellophane	
			Dumpsite, Pathum Thani	581.90 ± 28.39			PP, PU, PE, PS, and cellophane	
			Industrial estate, Samut Prakan	221.48 ± 31.58			PP, PE, and cellophane	
Breathing Thermal Manikin (indoor air, 24 h)	Transferring the sample to a new support material	Silver membrane 0.8 µm	Apartment in Aarhus, Denmark,	9.3 ± 5.8	Fragment and fiber	<11–237 µm	PES, PE, nylon, PP, and other polymers	[9]
Personal air sampler (indoor air, 8 h)	NR staining	GF/A filter 1.6 µm	Dormitory	849 ± 303	Fragment and fiber	<10 µm: 23.65–39.41%, 10–100 µm: 55.32–71.84%, 100–1000 µm: 3.91–7.24%, >1000: 0.18%	PET and PES	This study
			Laundry shop	470 ± 206			SAN	
			Office	192 ± 73			Cellophane	
			Van station	635 ± 199			SAN, PES, and cellophane	
Personal air sampler (outdoor air, 8 h)	NR staining	GF/A filter 1.6 µm	Chiang Rak Road	284 ± 155	Fragment and fiber	<10 µm: 30.44–47.83%, 10–100 µm: 50.18–61.05%, 100–1000 µm: 1.97–10.15%, >1000: 0.02–0.61%	PES and PE	This study
			WWTP	540 ± 136			PET	
			WSP in the University	3964 ± 2575			PP	
			WSP in the market	3474 ± 678			PS, EVA, SAN, and PET	

**Table 3:** Comparison of the personal intake of microplastics through inhalation and ingestion.

Exposure Pathway	Sample Group	Size	Recommended Estimated Consumption	Number of MPs	Daily Intake of MPs	References
Ingestion	Fruit and vegetables	<10 µm	400 g/day	132,740 n/g	53.09 × 10^6^	[33,34]
	Seafood	NA	22.41 kg/year	0.98 n/g	60.38	[33]
	Bottled water	<10–100 µm (>90% of MPs smaller than 10 µm)	2 L/day	13.55 × 10^6^ n/L	27.1 × 10^6^	[33,35]
	Salt	10–5000 µm	5 g/day	142.8 n/kg	0.71	[33,36]
	Alcohol	10–5000 µm	6.4 L/year	4.05 n/L	0.07	[33,36]
	Total consumption depends on an ingestion pathway of 80.19 × 10^6^ n/day					[33]
Inhalation (This study)	Housekeeper	<10 µm	6 L/min (8 h of working period)	312.1 n/m^3^	899	This study
	Laundromat staff			157.8 n/m^3^	454	
	Office worker			45.6 n/m^3^	131	
	Van driver			250.3 n/m^3^	721	
	Street vendor			86.4 n/m^3^	249	
	Maintenance technician in a WWTP			231.9 n/m^3^	668	
	Waste segregation officer in a university			1896.0 n/m^3^	5460	
	Waste segregation officer in market			1146.2 n/m^3^	3301	

## Data Availability

All data are reported in the present manuscript.

## Supplementary Materials

The following supporting information can be downloaded at: https://www.mdpi.com/article/10.3390/ijerph20247162/s1, Table S1: Data record of the housekeeper samples. Table S2: Data record of the laundromat staff samples. Table S3: Data record of the office worker samples. Table S4: Data record of the van driver samples. Table S5: Data record of the street vendor samples. Table S6: Data record of the maintenance technician in the WWTP samples. Table S7: Data record of the waste segregation officer in the university samples. Table S8: Data record of the waste segregation officer in the market samples.
