# Supplementary material for: Exploring Personal Exposure to Airborne Microplastics across Various Work Environments in Pathum Thani Province, Thailand
Source: Int J Environ Res Public Health. 2023 Dec 11;20(24):7162. doi: 10.3390/ijerph20247162 (PMC10743124; doi:10.3390/ijerph20247162)
Supplement: Supplementary file 1 [file ijerph-20-07162-s001.zip › ijerph-2691225-supplementary.pdf]

International Journal of
Environmental Research and Public Health

MDPI

Supplementary Materials

Exploring Personal Exposure to Airborne Microplastics across Various Work Environments in Pathum Thani Province, Thailand

Kanokwan Limsiriwong and Ekbordin Winijkul *

Environmental Engineering and Management, Asian Institute of Technology, Pathum Thani 12120, Thailand; kanokwan@ait.asia
* Correspondence: ekbordinw@ait.asia

Table S1. Data Record of the Housekeeper Samples.

**Information**		Housekeeper			
**Samples**		H_blank	H1	H2	H3
**Date**			14/12/2022	15/12/2022	16/12/2022
**1. Location**		Asian Institute of Technology			
**2. Specific area**		Dormitory F			
**3. No. of devices**		2			
**4. Sex**		Male			
**5. Career**		Housekeeper			
**6. Daily Activity/Environment condition**					
The housekeeper cleans eight student rooms per day, such as sweeping and mopping floors, cleaning windows, and washing balconies. These rooms have no air conditioner. All rooms have the same size and furniture, such as a bed, wardrobe, desk, and shelf. However, the amount of individual stuff in each room varies, such as books, clothes, and miscellanies.					
**7. Flow rate (L/min)**		2			
**8. Sampling period**	Start (a.m.)		7.42	7.42	7.50
	Stop (p.m.)		15.42	15.42	15.50
	Total time(min)		480	480	480
**9. Environmental parameters**	Temperature (℃)		20	20	21
	Humidity (%)		77	75	78
	Pressure (hPa)		1014	1013	1012
**10. Sampling Air Volume (L)**			960	960	960
**11. Weight of filter samples (g)**	W1 (g); after	0.14677	0.13732	0.14809	0.14788
	W2 (g); before	0.14677	0.13706	0.14779	0.14767
	The weight of total particle(g)	0	0.00026	0.0003	0.00021
	The weight of total particle ×1000 (mg)	0	0.26	0.3	0.21
**12. TSP Concentration (mg/m³)**			0.271	0.312	0.219
**13. Total AMPs (pieces)**		6	479	973	993
**14. The number of AMPs (n/m³)**			499	1014	1034

Table S2. Data Record of the Laundromat Staff Samples.

Information		Laundromat staff			
**Samples**		L_blank	L_1	L_2	L_3
**Date**			21/12/2022	22/12/2022	23/12/2022
**1. Location**		Laundry Shop in Thammasat Dormitory			
**2. Specific area**		Thammasat University (Rangsit campus)			
**3. No. of devices**		2			
**4.Sex**		Female			
**5. Career**		Laundromat staff			
**6. Daily Activity/Environment condition**					
The laundromat staff has daily activities, such as ironing and using washing machines, constantly monitoring and checking the machines. Within the workplace, there are five washing machines, and the number of clothes that were washed was around 50-60 pieces per day.					
**7. Flow rate (L/min)**		2			
**8. Sampling period**	Start (a.m.)		9.15	9.11	9.06
	Stop (p.m.)		17.15	17.11	17.06
	Total time(min)		480	480	480
**9. Environmental parameters**	Temperature (°C)		25	25	25
	Humidity (%)		63	65	71
	Pressure (hPa)		1013	1013	1013
**10. Sampling Air Volume (L)**			960	960	960
**11. Weight of filter samples (g)**	W1 (g); after	0.15025	0.15421	0.15619	0.14845
	W2 (g); before	0.15025	0.15412	0.1561	0.14831
	The weight of total particle(g)	0	0.00009	0.00009	0.00014
	The weight of total particle ×1000 (mg)	0	0.09	0.09	0.14
**12. TSP Concentration (mg/m³)**			0.094	0.094	0.146
**13. Total AMPs (pieces)**		8	328	347	680
**14. The number of AMPs (n/m³)**			342	361	708

Table S3. Data Record of the Office Worker Samples.

**Information**		**Office worker**			
**Samples**		AIT_blank	AIT_1	AIT_2	AIT_3
**Date**			20/1/2023	23/1/2023	24/1/2023
**1. Location**		Asian Institute of Technology			
**2. Specific area**		SERD Visiting Office E-113			
**3. No. of devices**			2	1	2
**4. Sex**		Female			
**5. Career**		AIT Officer			
**6. Daily Activity/Environment condition**					
The office worker spent time at the personal desk to do paperwork and coordinate in the private room. The room has is an air-conditioned room and includes basic office equipment such as desks, chairs, computers, and printers.					
**7. Flow rate (L/min)**		2			
**8. Sampling period**	Start (a.m.)		9.30	8.43	8.50
	Stop (p.m.)		17.30	16.43	16.50
	Total time(min)		480	480	480
**9. Environmental parameters**	Temperature (℃)		26	25	26
	Humidity (%)		60	62	58
	Pressure (hPa)		1016	1012	1013
**10. Sampling Air Volume (L)**			960	960	960
**11. Weight of filter samples (g)**	W1 (g); after	0.14859	0.14576	0.15123	0.14947
	W2 (g); before	0.14859	0.14569	0.15119	0.14944
	The weight of total particle(g)	0.00000	0.00007	0.00004	0.00003
	The weight of total particle ×1000 (mg)	0	0.07	0.04	0.03
**12. TSP Concentration (mg/m³)**			0.073	0.042	0.031
**13. Total AMPs (pieces)**		4	110	195	249
**14. The number of AMPs (n/m³)**			115	203	259

Table S4. Data Record of the Van Driver Samples.

**Information**		Van Driver			
**Samples**		VD_blank	VD1	VD2	VD3
**Date**			7/2/2023	8/2/2023	10/2/2023
**1. Location**		Van station (TU-Victory Monument)			
**2. Specific area**		Thammasat University (Rang-sit campus)			
**3. No. of devices**		2			
**4.Sex**		Male			
**5. Career**		Van Driver			
**6. Daily Activity/Environment condition**					
The daily activity of the van driver is driving approximately 4-5 trips per day from Pathum Thani to Bangkok (around 1-2 hours per trip). The van has a capacity of 14 seats, and the seating material is made from synthetic textiles.					
**7. Flow rate (L/min)**		2			
**8. Sampling period**	Start (a.m.)		8.4	9.03	8.54
	Stop (p.m.)		18.36	19.16	18.26
	Total time(min)		599	613	573
**9. Environmental parameters**	Temperature (°C)		28	29	29
	Humidity (%)		77	76	78
	Pressure (hPa)		1012	1012	1012
**10. Sampling Air Volume (L)**			1198	1226	1146
**11. Weight of filter samples (g)**	W1 (g); after	0.14377	0.14972	0.15223	0.14927
	W2 (g); before	0.14377	0.14965	0.15215	0.14919
	The weight of total particle(g)	0.00000	0.00007	0.00008	0.00008
	The weight of total particle ×1000 (mg)	0	0.07	0.08	0.08
**12. TSP Concentration (mg/m³)**			0.058	0.065	0.070
**13. Total AMPs (pieces)**		29	909	497	850
**14. The number of AMPs (n/m³)**			759	405	742

Table S5. Data Record of the Street Vendor Samples.

**Information**		**Street Vendor**			
**Samples**		V_blank	V1	V2	V3
**Date**			6/12/2022	8/12/2022	9/12/2022
**1. Location**		Chiang Rak Road			
**2. Specific area**		Thammasat University gate1(Rang-sit campus)			
**3. No. of devices**		1			
**4.Sex**		Female			
**5. Career**		Street Vendor			
**6. Daily Activity/Environment condition**					
The daily activities are cooking and selling food. The shop was at the roadside (1 meter away from the road), and cars have been passing the shop all day.					
**7. Flow rate (L/min)**		2			
**8. Sampling period**	Start (a.m.)		7.00	7.15	7.20
	Stop (p.m.)		15.00	15.15	15.20
	Total time(min)		480	480	480
**9. Environmental parameters**	Temperature (℃)		30	30	31
	Humidity (%)		68	65	64
	Pressure (hPa)		1013	1012	1011
**10. Sampling Air Volume (L)**			960	960	960
**11. Weight of filter samples (g)**	W1 (g); after	0.15086	0.14902	0.1429	0.14591
	W2 (g); before	0.15086	0.14873	0.14254	0.14553
	The weight of total particle(g)	0	0.00029	0.00036	0.00038
	The weight of total particle ×1000 (mg)	0	0.29	0.36	0.38
**12. TSP Concentration (mg/m³)**			0.302	0.375	0.396
**13. Total AMPs (pieces)**		21	185	445	188
**14. The number of AMPs (n/m³)**			193	464	196

Table S6. Data Record of the Maintenance Technician in WWTP Samples.

**Information**		**Maintenance technician in WWTP**			
**Scheme 1.**		WWTP blank	WWTP1	WWTP2	WWTP3
**Date**			9/1/2023	10/1/2023	11/1/2023
**1. Location**		Wastewater treatment plant			
**2. Specific area**		Thammasat University (Rang-sit campus)			
**3. No. of devices**		2			
**4.Sex**		Male			
**5. Career**		Maintenance technician			
**6. Daily Activity/Environment condition**					
The wastewater treatment plant consists of an aeration tank (surface area of 5000 m^2), a sedimentation tank (surface area of 2600 m^2), and a chlorine tank (surface area of 87.5 m^2). The plant operates with an aeration system and receives approximately 1500 m^3/day of wastewater from the university. The staff is responsible for maintenance and regular inspections of the treatment system.					
**7. Flow rate (L/min)**		2			
**8. Sampling period**	Start (a.m.)		8.14	8.15	8.15
	Stop (p.m.)		16.14	16.15	16.15
	Total time(min)		480	480	480
**9. Environmental parameters**	Temperature (°C)		27	25	24
	Humidity (%)		61	78	87
	Pressure (hPa)		1015	1014	1013
**10. Sampling Air Volume (L)**			960	960	960
**11. Weight of filter samples (g)**	W1 (g); after	0.15569	0.17078	0.16365	0.17937
	W2 (g); before	0.15569	0.17063	0.16357	0.17927
	The weight of total particle(g)	0.00000	0.00015	0.00008	0.00010
	The weight of total particle ×1000 (mg)	0	0.15	0.08	0.10
**12. TSP Concentration (mg/m³)**			0.156	0.083	0.104
**13. Total AMPs (pieces)**		6	387	648	520
**14. The number of AMPs (n/m³)**			403	675	542

Table S7. Data Record of the Waste Segregation Officer in The University samples.

**Information**		**Waste segregation officer in the university**			
**Samples**		WSPTU blank	WSPTU1	WSPTU2	WSPTU3
**Date**			17/1/2023	18/1/2023	19/1/2023
**1. Location**		Waste separation Plant			
**2. Specific area**		Thammasat University (Rang-sit campus)			
**3. No. of devices**		2			
**4.Sex**		Female			
**5. Career**		Waste segregation officer			
**6. Daily Activity/Environment condition**					
The duty of this officer is to segregate recycled waste from the general waste. This waste segregation plant receives municipal solid wastes (organic and plastic wastes) approximately 6-7 tons per day from the university. Plastic waste accounts for approximately 40-50% of all waste, mostly consisting of PP and PE from plastic bags and plastic bottles.					
**7. Flow rate (L/min)**		2			
**8. Sampling period**	Start (a.m.)		8.10	8.03	8.19
	Stop (p.m.)		16.10	16.03	16.19
	Total time(min)		480	480	480
**9. Environmental parameters**	Temperature (℃)		28	24	23
	Humidity (%)		69	74	73
	Pressure (hPa)		1014	1016	1016
**10. Sampling Air Volume (L)**			960	960	960
**11. Weight of filter samples (g)**	W1 (g); after	29.12658	28.67735	29.54511	28.01981
	W2 (g); before	29.12658	28.67726	29.54472	28.01962
	The weight of total particle(g)	0.00000	0.00009	0.00039	0.00019
	The weight of total particle ×1000 (mg)	0	0.09	0.39	0.19
**12. TSP Concentration (mg/m³)**			0.094	0.406	0.198
**13. Total AMPs (pieces)**		4	6498	1640	3278
**14. The number of AMPs (n/m³)**			6769	1708	3415

Table S8. Data Record of the Waste Segregation Officer in The Market Samples.

**Information**		**Waste segregation officer in the market**			
**Samples**		WSP4 blank	WSP4_1	WSP4_2	WSP4_3
**Date**			30/1/2023	31/1/2023	1/2/2023
**1. Location**		Waste separation Plant			
**2. Specific area**		Simummuang Market			
**3. No. of devices**		2			
**4.Sex**		Male			
**5. Career**		Waste segregation officer			
**6. Daily Activity/Environment condition**					
The waste separation plant in the market only deals with recyclable waste, such as paper, glass bottles, plastic bottles, plastic bags, and plastic trays. The recyclable plastic is predominantly made from PE and PP materials. The staff's responsibility is to segregate the types and colors of plastic trays before crushing them into small sizes with a blender. This process facilitates the easy transport of plastics for recycling into new trays. The waste segregation plant receives approximately 1 ton/day of plastic trays from the market. Additionally, this plant also gets plastic bottles and plastic bags around 2 tons/day. They have equipment to rinse and break down plastic bags into smaller pieces. The plastics are then laid on the ground to dry. Therefore, the staff must turn the plastic bags from the bottom to the top to ensure they are dry before sending them for the recycling process.					
**7. Flow rate (L/min)**		2			
**8. Sampling period**	Start (a.m.)		7.40	7.42	7.54
	Stop (p.m.)		15.40	15.42	15.54
	Total time(min)		480	480	480
**9. Environmental parameters**	Temperature (°C)		18	18	24
	Humidity (%)		61	63	61
	Pressure (hPa)		1016	1013	1012
**10. Sampling Air Volume (L)**			960	960	960
**11. Weight of filter samples (g)**	W1 (g); after	29.10273	28.65721	29.52189	27.99651
	W2 (g); before	29.10272	28.6568	29.52136	27.99552
	The weight of total particle(g)	0.00001	0.00041	0.00053	0.00099
	The weight of total particle ×1000 (mg)	0.01	0.41	0.53	0.99
**12. TSP Concentration (mg/m³)**			0.427	0.552	1.031
**13. Total AMPs (pieces)**		48	4073	2846	3085
**14. The number of AMPs (n/m³)**			4243	2965	3214
